# IBUPROFEN-INDUCED ASEPTIC MENINGITIS: A CASE REPORT

**DOI:** 10.1590/1984-0462/;2019;37;3;00016

**Published:** 2019-06-03

**Authors:** Sofia Alexandra Pereira Pires, Ana Pereira Lemos, Ester Preciosa Maio Nunes Pereira, Paulo Alexandre da Silva Vilar Maia, João Patrício de Sousa e Alvim Bismarck do Agro

**Affiliations:** a Centro Hospitalar e Universitário de Coimbra, Coimbra, Portugal.; b Centro Hospitalar de Leiria, Leiria, Portugal.

**Keywords:** Meningitis, aseptic, Anti-inflammatory agents, non-steroidal, Ibuprofen, Adolescent, Meningite asséptica, Anti-inflamatórios não esteroides, Ibuprofeno, Adolescente

## Abstract

**Objective:**

To report a case of a male adolescent with the diagnosis of ibuprofen-induced meningitis. We discuss themain causes of drug-induced aseptic meningitis (DIAM) and highlight the importance of early recognition of DIAM, sothat the offending drug can be withdrawn, and recurrences prevented. Only few DIAM cases have been reported in pediatric age.

**Case description:**

A healthy 15-year-old boy presented to the emergency department with headache, nausea, dizziness, fever, conjunctival hyperemia and blurred vision 30 minutes after ibuprofen-intake. During his stay, he developed emesis and neck stiffness. Cerebrospinal ﬂuid analysis excluded infectious causes, and DIAM was considered. He totally recovered after drug withdrawal.

**Comments:**

DIAM is a rare entity, that should be considered in the differential diagnosis of an aseptic meningitis. The major causative agents are nonsteroidal anti-inflammatory drugs, particularly ibuprofen. Suspicion is made by the chronologic link between drug intake and the beginning of symptoms, but infectious causes should always be ruled out.

## RESUMO

**Objetivo:**

Descreve-se o caso de um adolescente do sexo masculino com diagnóstico de meningite asséptica por ibuprofeno. Discutem-se as causas de meningite asséptica induzida por medicamentos (MAIM) e a importância do reconhecimento precoce dessa situação, para que a medicação envolvida seja suspensa e as recorrências prevenidas. Poucos casos foram descritos em idade pediátrica.

**Descrição do caso:**

Adolescente de 15 anos, gênero masculino, saudável, procurou o serviço de urgência por cefaleia, náuseas, tonturas, febre, hiperemia conjuntival e visão desfocada 30 minutos após o uso de ibuprofeno. Durante a internação, iniciou vômitos e rigidez na nuca. A análise do líquido cefalorraquidiano excluiu causas infeciosas, e considerou-se como diagnóstico mais provável a MAIM. A recuperação foi total após a suspensão do medicamento.

**Comentários:**

A MAIM é rara, mas deve ser considerada no diagnóstico diferencial de meningite asséptica. A principal causa são os anti-inflamatórios não esteroides, principalmente o ibuprofeno. A suspeita clínica é evocada pela relação temporal entre o uso do medicamento e o início dos sintomas, mas as causas infeciosas devem ser sempre excluídas.

## INTRODUCTION

Aseptic meningitis is a nonbacterial inflammation of the meninges.^1^ Most commonly, it is caused by virus infection, but a variety of infectious and noninfectious agents, including drugs, can be implied.^2^

Drug-induced aseptic meningitis (DIAM) is a rare entity and constitutes a diagnosis and patient management challenge. The most commonly responsible drugs are nonsteroidal anti-inflammatory drugs (NSAIDs), antibiotics, immunosuppressive-immunomodulatory, antiepileptic and intrathecal drugs.^3^ ^,^ ^4^

Ibuprofen is the main cause of DIAM, having been described in several case reports, especially in adults with autoimmunedisorders.^4^ ^,^ ^5^ ^,^ ^6^

## CASE DESCRIPTION

A previously healthy 15-year-old male presented to the emergency departmentwith headache, nausea, dizziness, fever andblurred vision occurring 30 minutes after taking a single dose of ibuprofen 400 mg. A week earlier, he also had headache, nausea and fever during one day after ibuprofen-intake, prescribed due to a fracture of the fifth right metatarsal bone.

There was a family history of autoimmune disease. His20-year-old sister had autoimmune thyroiditis.

On examination, hewas febrile (auricular temperature of 38.4ºC), with blood pressure 130/71 mmHg and pulse rate 93/minute. His neurological exam was normal with no altered mental status nor meningeal or focal signs. He presented conjunctival hyperemia, without ocular discharge. Other organ systems’ examination was unremarkable.

Laboratory tests revealed total white cell count 9,900/µL,with a differential of 76,9% neutrophils and 19,4% lymphocytes, C-reactive protein 1.2 mg/L and negativeblood culture.

He was admitted for clinical surveillance. On day 1, the patient maintained fever, and headache became more intense, with emesis. On examination, he had neck stiffness. Lumbar puncture was performed and cerebrospinal ﬂuid (CSF) analysis revealed a clear ﬂuid, with total white cell count 268/mm^3^ (0% neutrophils and 100% lymphocytes), glucose 58 mg/dL and total protein 720 mg/dL. No organisms were seen on gram stain and culture was negative. Screening for bacterial antigens(*Haemophilus influenzae* type b, *Streptococcus pneumoniae*, *Neisseria meningitidis* A, B and C, and *Escherichia coli k1*) andenterovirus polymerase chain reaction (PCR) were also negative. Ibuprofen was discontinued, and the patient improved without specific treatment. Symptoms resolved within 48 hours after admission, with no neurologic sequelae.

The patient was discharged with a presumptivediagnosis of aseptic meningitis induced by ibuprofen. He was advised not to takeNSAIDs. Screening for autoimmune diseases was negative and included antinuclear, antiphospholipid and antithyroid antibodies.

A report to the National Authority of Medicines and Health Products (Infarmed) was made, that considered probable the link between ibuprofen intake and aseptic meningitis.

## DISCUSSION

NSAIDs are the most common drugs implicated in DIAM.^7^ ^,^ ^8^ The first report was made in 1978, in a patient with systemic lupus erythematosus (SLE) who had an ibuprofen-induced meningitis.^9^Since then, several reports of aseptic meningitis attributed to NSAIDs have been made, including dexibuprofen, dexketoprofen, flurbiprofen, diclofenac, sulindac, ketoprofen, tolmetin, piroxicam, indometacin, naproxen and prostaglandin-endoperoxide synthase 2 (COX-2) inhibitors (rofecoxib and celecoxib).^5^ ^,^ ^8^ ^,^ ^10^ Ibuprofen is, nevertheless, by far the leading cause of DIAM.^5^

To our knowledge, there are only two published case reports of NSAIDs-induced aseptic meningitis that presented at pediatric age, caused by ibuprofen^3^ and rofecoxib,^11^ respectively. We summarize clinical and laboratorial features on Table 1.

**Table 1:** Clinical and laboratorial features of three pediatric patients with nonsteroidal anti-inflammatory drugs-induced meningitis.

	Bonnel et al. 2002^11^	Frank-Briggs et al. 2014^3^	Pires et al. 2019*
Age	16	15	15
Gender	Female	Male	Male
Drug (dose)	Rofecoxib (12,5 mg/day)	Ibuprofen (not available)	Ibuprofen (400 mg/dose)
Associated diseases	None	None	None
Time from drug intake and symptoms	12 days	3 days	30 minutes
Symptoms	Headache, numbness in the right leg and right side of the face, altered mental status	Fever (39ºC), altered mental status, positive Kerning sign, hypertonia of the lower limbs	Fever (38,4ºC), conjunctival hyperemia, headache, dizziness, blurred vision, emesis, neckstiffness
CSF			
- White blood cells/mm^3^	- 82	- 75	- 268
- Neutrophils (%)	- 0	- 27	- 0
- Lymphocytes (%)	- 100	- 73	- 100
- Glucose (mg/dL)	- Not available	- 42	- 58
- Proteins (mg/dL)	- Increased (no values available)	- 102	- 720
- Cultures	- Negative	- Negative	- Negative
- Other	- Negative for viral, fungal and bacterial isolates	- Negative CMV, HSV 1 e 2, EBV, arbovirus and Mycobacterium tuberculosis PCR	- Negative enterovirus PCR
Antibiotic treatment	Ceftriaxone and acyclovir	Vancomycin and ceftriaxone	None
Sequels	None	None	None

CSF: cerebrospinal ﬂuid; CMV: cytomegalovirus; HSV: herpes simplex virus; EBV: Epstein-Barr virus; PCR: polymerase chain reaction. *This study.

Usually, patients with DIAM present with fever, altered mental status, headache, neck stiffness, nausea and vomiting. However, meningoencephalitis with neurological focal deficits is not infrequent. Blurred vision and conjunctivitis may occur, as in our case report.^4^ ^,^ ^5^ ^,^ ^6^

DIAM is a diagnosis of exclusion and infectious causes must always be ruled out. Clinical presentation may be quite similar to acute bacterial meningitis and CSF analysis doesn’t allow the exclusion of this diagnose until the culture is negative. CSF findings are not specific, and typically there is pleocytosis with neutrophilic predominance (72,2% of cases), normal-to-low glucose values, and increased proteins.^5^ Therefore, if there is a clinical suspicion of bacterial meningitis, antibiotic therapy should be given until negativity of cultures. Also, in cases that present as meningoencephalitis, antiviral treatment should be considered.

Occasionally, lymphocytes and even eosinophils may predominate.^5^ ^,^ ^12^ In our patient, however, we found pleocytosis with 100% lymphocytes, increased proteins and normal glycorrhachia.^13^ Some authors suggest that different results in CSF can be related to the timing of which the lumbar puncture is done, corresponding to different stages of the disease.^10^ ^,^ ^14^ ^,^ ^15^ Normalization of CSF parameters occurs quite slowly, over several days.^5^ ^,^ ^12^

On the other hand, viral meningitis may be quite difficult to differentiate from DIAM. In our case report, and according to clinical manifestations and CSF findings, we did CSF enterovirus PCR to exclude infection by this main viral agent.^1^

DIAM is suspected by the establishment of a temporal relationship between the administration of the drug and the onset of symptoms. Latency period is short, typically less than 48 hours.^3^ There is a rapid resolution of symptoms after drug withdrawal, one to five days.^4^ ^,^ ^12^ On the contrary, in viral meningitis, time to recovery is longer, 10 to 14 days.^6^

DIAM can occur in patients that previously tolerated the offending drug.^3^ ^,^ ^6^ Prior exposures to the drug have been noted in 35% of patients with ibuprofen-induced meningitis.^4^ In our patient, a shorter period between ibuprofen-intake and the onset of symptoms and increased symptoms’ severity during the second exposure were helpful clues for the diagnosis.Moreover, the adolescent also completely recovered only 48 hours after drug withdrawal.

DIAM is more frequent in women (64%) and recurrent episodes are quite frequent (61%), especially when the diagnosis is not made on the first one.^5^ ^,^ ^15^ ^,^ ^16^ Drug challenge can confirm diagnosis,^14^ but it is not advised, as re-exposure can lead to severe symptoms.^17^

Patients must be advised to avoid not only ibuprofen, butalso all NSAIDs, because recurrent episodes of aseptic meningitis related to other NSAIDs can occur.^10^ ^,^ ^18^ We should provide medical alternatives to the patient and explain that aseptic meningitis is independent of the dose of NSAID.^5^

There is a strong association between DIAM and autoimmune diseases (specially SLE), so it is recommended an analytical screening even in asymptomatic patients.^5^ ^,^ ^17^ Thehigher incidence of ibuprofen-induced aseptic meningitis in patients with autoimmune diseases could be related to the widespread use of NSAIDs or by patients’ tendency to autoreact.^5^ ^,^ ^15^ Inpediatric population, this is quite important, because DIAM can potentially be the first sign that leads to the suspicion of an underlying autoimmune disorder. In our case report, despite family history of autoimmune disease, autoantibodies were negative.

The mechanism by which NSAIDs cause meningeal inflammation is uncertain, but it appears to be an immunologically mediated hypersensitivity reaction (type III or IV).^1^ ^,^ ^5^ ^,^ ^6^

The increasing number of NSAIDs-induced aseptic meningitis, even in healthy people, can be related to the broader use of this medication.^5^ This could be a greater problem in countries where they are purchased over-the-counter, like in Portugal. So, it is very important to arise medical attention for this condition and to report drug side effects.

Our case report of aseptic meningitis due to ibuprofen intake illustrates the importance of early diagnosis, so that the offending drug can be withdrawal and recurrences prevented. A rapid onset and resolution of symptoms with compatible CSF finding suggest DIAM. As DIAM is a diagnosis of exclusion, it is important to investigate and exclude other causes of meningitis, especially infectious agents, and always obtain a detailed drug history.^12^ The prognosis is usually good, with no long-term sequels.^5^
